# Vaccination intentions of hypertensive Chinese individuals during the COVID-19 epidemic: a structural equation modeling study

**DOI:** 10.1186/s12879-024-09480-0

**Published:** 2024-06-26

**Authors:** Zhi Lei, Dongyang Liu, Minghui Li, Deqiang Xian, Song Fan

**Affiliations:** 1 Department of Chronic Disease Control and Prevention, Luzhou Center for Disease Control and Prevention, Luzhou, China; 2 School of Public Health, Southwest Medical University, Luzhou, China; 3 Department of Human Resource, Sichuan Provincial Center for Disease Control and Prevention, Chengdu, China; 4 Administrative Office, Luzhou People’s Hospital, Luzhou, China

**Keywords:** Hypertension, COVID-19, Vaccination, Structural equation modeling

## Abstract

**Objective:**

Given the high prevalence of hypertension among Chinese adults, this population is at a significantly increased risk of severe COVID-19 complications. The purpose of this study is to assess the willingness of Chinese hypertensive adults to receive the COVID-19 vaccine and to identify the diverse factors that shape their vaccination decisions.

**Methods:**

Sampling was conducted utilizing multistage stratified random sampling, and ultimately, a total of 886 adult hypertensive patients from Luzhou City in Southwest China were included in this study. The questionnaire design was based on the Theory of Planned Behaviour and was used to investigate their willingness to be vaccinated with COVID-19. Structural equation modeling was employed for data analysis.

**Results:**

The results showed that 75.6% of hypertensive individuals were willing to receive COVID-19 vaccination. The structural equation modeling revealed that Subjective Norms (path coefficient = 0.361, CR = 8.049, *P* < 0.001) and Attitudes (path coefficient = 0.253, CR = 4.447, *P* < 0.001) had positive effects on vaccination willingness, while Perceived Behavioral Control (path coefficient=-0.004, CR=-0.127, *P* = 0.899) had no significant impact on Behavioral Attitudes. Mediation analysis indicated that Knowledge (indirect path coefficient = 0.032, LLCI = 0.014, ULCI = 0.058), Risk Perception (indirect path coefficient = 0.077, LLCI = 0.038, ULCI = 0.124), and Subjective Norms (indirect path coefficient = 0.044, LLCI = 0.019, ULCI = 0.087) significantly influenced vaccination willingness through Attitudes as a mediating factor.

**Conclusion:**

The willingness of hypertensive individuals to receive the COVID-19 vaccination is not satisfactory. The Theory of Planned Behavior provides valuable insights into understanding their vaccination intentions. Efforts should be concentrated on enhancing the subjective norms, attitudes, and knowledge about vaccination of hypertensive patients.

## Introduction

The COVID-19 pandemic has had a significant impact on public health worldwide. Hypertension is one of the underlying health conditions that increases the risk of severe illness and death from COVID-19. The number of people who living with hypertension is huge in China, and several studies have shown that hypertension is a critical risk factor for COVID-19 infection and disease severity [1–3]. Therefore, it is crucial to identify effective strategies to prevent and control COVID-19 in this vulnerable population.

Vaccination is a crucial tool in reducing the morbidity and mortality associated with COVID-19. However, vaccine hesitancy remains a significant obstacle in achieving high vaccination coverage rates in some populations, including hypertensive individuals [4, 5]. The theory of planned behavior has been widely used in various studies examining vaccination intention [6–8]. This theory proposes that an individual’s attitudes, subjective norms, and perceived behavioral control significantly impact their willingness to vaccinate [9].

Given the importance of vaccination in preventing COVID-19, it is essential to investigate the factors that influence hypertensive individuals’ willingness to receive the COVID-19 vaccine. Conducting research based on the theory of planned behavior can provide valuable insights into the underlying reasons for vaccine hesitancy and identify potential barriers to vaccination. The findings of this study can inform the development of effective strategies to increase COVID-19 vaccination coverage rates in the hypertensive population, thus reducing the impact of the pandemic.

Briefly, this study sought to determine the willingness of Chinese hypertensive patients to receive the COVID-19 vaccine, utilizing the Theory of Planned Behaviour to identify the factors that shape their decisions. The ultimate objective is to develop a vaccination strategy more tailored to suit the specific needs of this population.

## Subjects and methods

### Participants

In this study, a questionnaire survey was conducted among people over 18 years old with hypertension in Southwest China - Luzhou from May to June 2022. Multi-stage stratified sampling was utilized to survey hypertensive patients across seven districts in the city. Stratification was based on urban and rural classifications, and within each county or district, three towns or communities were randomly selected for the survey. This study was approved by the Ethics Review Committee of Luzhou Center for Disease Control and Prevention (No. 2,021,001).

Based on our pre-survey indicating a 76.6% willingness to receive the COVID-19 vaccine among hypertensive patients, along with a projected non-response rate of 20%, a confidence level of 95% (corresponding to a z-score of 1.96), and a margin of allowable error of d = 0.05, we calculated that a minimum sample size of 345 participants was necessary for our study. Finally, a total of 933 individuals participated in the survey. However, 47 participants were excluded due to contraindications to the vaccine, such as pregnancy, uncontrolled blood pressure medication, diabetes mellitus, and other factors. As a result, a total of 886 valid questionnaires were included in the study.

Inclusion criteria were: (1) aged 18 years or above; (2) diagnosed with hypertension and blood pressure is within the control of medication; (3) able to communicate effectively and voluntarily participate in the survey. Exclusion criteria were: (1) have any contraindications to Covid-19 vaccination; (2) unable to understand the content of the questionnaire.

### Procedure

This cross-sectional study was conducted using a self-administered questionnaire, and survey data Survey data was collected online using an online survey tool named Wenjuanxing. The questionnaire was developed based on the Theory of Planned Behavior (TPB) and previous studies on vaccination intention. Before the formal survey, a pre-survey was conducted with 30 participants to examine the clarity and feasibility of the questionnaire. The results of the pre-survey were used to modify and improve the questionnaire. The final version of the questionnaire consisted of three parts.

Part 1 General demographic information, including gender, age, education, marital status, and occupation.

Part 2 The TPB model measures several variables, specifically subjective norm, perceived behavior control and behavioral attitude. Subjective norm was assessed through four items, like “I would be more willing to vaccinate if recommended by medical staff.” Perceived behavior control was evaluated using three items, such as “Is it convenient to make an appointment for COVID-19 vaccination?” Behavioral attitude was gauged through two items, including “I believe the vaccine provides effective protection against COVID-19.” These survey items were all rated on a five-point Likert scale, ranging from strongly disagree (1) to strongly agree (5). The total scores of the respective items constituted the final score for each dimension, and the cutoff points used for classifying each of these variables was based on the median score. Additionally, a risk perception dimension encompassing two inquiries, such as “If I contract pneumonia, it will increase the burden on me and my family.” Responses to these inquiries were also rated on a five-point Likert scale, spanning from strongly disagree (1) to strongly agree (5). Beyond this, the study also introduced a knowledge dimension was integrated into the TPB framework, encompassing four items designed to test participants’ understanding of COVID-19 and the vaccine. Each correct response earned one point, and the cumulative total represented the overall knowledge score.

Part 3 The willingness of participants to get vaccinated against COVID-19. Two items were used to measure vaccination intention, such as “Are you willing to receive the COVID-19 vaccine?” Responses to both items are rated using a 5-point Likert scale, ranging from strongly disagree (1) to strongly agree (5), and the overall vaccination intention score is determined by averaging the scores of these two items. Scores of 3 or above were categorized as “willing to vaccinate”, while scores below 3 were deemed as “unwilling to vaccinate”.

### Data analysis

Data were analyzed using Microsoft Excel 2019 for data cleaning and descriptive statistics. Statistical analysis was conducted using SPSS 26.0. A structural equation model was built using AMOS 24 software to test the relationship between vaccination intention and the TPB variables, including behavioral attitude, subjective norm, and perceived behavior control. The model fit was assessed using several fit indices, including the chi-square test, comparative fit index (CFI), root mean square error of approximation (RMSEA), and standardized root mean square residual (SRMR). The statistical significance was defined as *p* < 0.05. Hypotheses(H1-H7) were tested using regression analysis, and the effect size was estimated using standardized regression coefficients (β). Following assumptions were proposed:

H1: Knowledge about COVID-19 vaccination was positively correlated with attitude towards COVID-19 vaccination.

H2: Risk perceptions of COVID-19 are positively correlated with attitude towards COVID-19 vaccination.

H3: Subjective norm was positively correlated with the attitude towards COVID-19 vaccination.

H4: Perceptual behavior control was positively correlated with the attitude towards COVID-19 vaccination.

H5: Subjective norm was positively correlated with the vaccination intention against COVID-19.

H6: Attitude towards COVID-19 vaccination will be positively correlated with the vaccination intention against COVID-19.

H7: Perceptual behavior control was positively correlated with the vaccination intention against COVID-19.

**Fig. 1:**
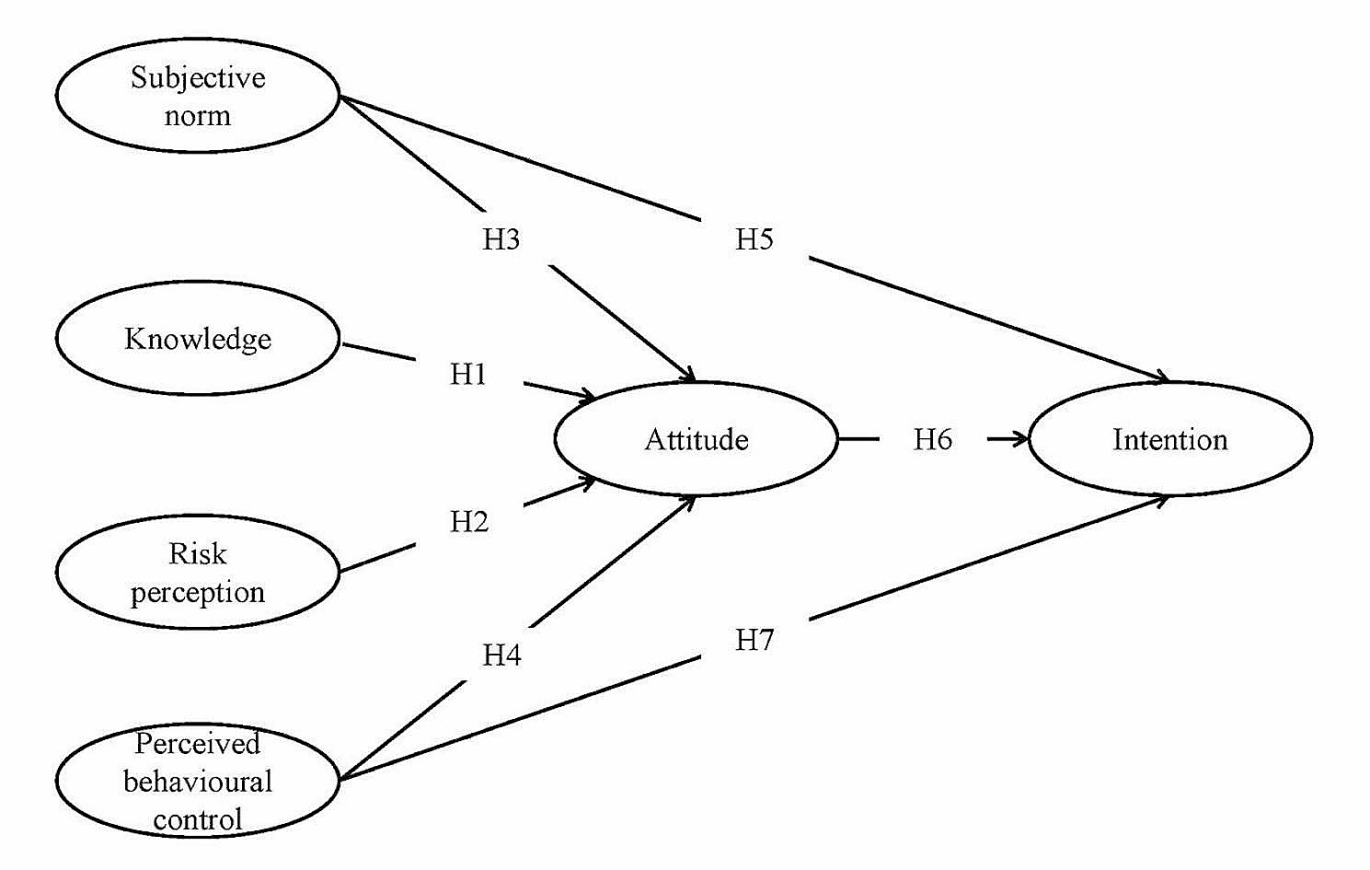
The proposed model in explaining COVID-19 vaccination uptake among people with hypertension

Based on these 7 assumptions above, we developed a structural equation model (Fig. 1). Knowledge, risk perception, subjective norms and perceived behavioural control influence study outcome (COVID-19 vaccination intention) by influencing attitudes, meanwhile, subjective norms and perceived behavioural control can directly influence outcome. In addition, using a bootstrap approach to test for indirect effects, we found two significant mediating pathways were identified in the model: (i) knowledge → attitude → COVID-19 vaccination intention pathway, and (ii) risk perception → attitude → COVID-19 vaccination intention pathway.

## Results

### Basic information

The 886 valid study participants included in this study included 474 (53.5%) males and 412 (46.5%) females; the average age was 64(10.2) years; and 713 (80.5%) had junior secondary education or below. Regarding marital status, 785 (88.6%) were married; 727 (82.1%) had an average monthly income of less than RMB 3,000.

The willingness of people with hypertension to vaccination was 75.6% (670/886), in which 76.6% and 74.5% for men and women respectively, with no statistically significant difference. The difference between ages was statistically significant (*p* < 0.01), with the highest willingness to vaccinate in the 21–44 age group (92.9%) and the lowest willingness to vaccinate in the 60 + age group (71.5%). In terms of marital status, the willingness to vaccinate was higher among the married group than among the unmarried/divorced /widowed (*p* < 0.05). As for the education level as well as the average monthly income, there was no significant difference between the groups. In terms of Attitude, Perceived behavioural control and Subjective norm, the willingness to vaccinate was higher in the high score group than in the low score group (*p* < 0.01 in each group). See Table 1.

**Table 1:** Participants’ characteristics

Characteristic	*N*(%)	Vaccination Intention		χ2	Ρ
		Yes	No		
**Gender**					
Male	474(53.5)	363(76.6)	111(23.4)	0.51	0.475
Female	412(46.5)	307(74.5)	105(25.5)		
**Age**				22.82	0.000
18–44	56(6.3)	52(92.9)	4(7.1)		
45–59	187(21.1)	158(84.5)	29(15.5)		
≥ 60	643(72.6)	460(71.5)	183(28.5)		
**Education**				5.75	0.057
Junior high school and below	713(80.5)	530(74.3)	183(25.7)		
High school/Vocational high school/Specialized secondary school	103(11.6)	79(76.7)	24(23.3)		
Junior college and above	70(7.9)	61(87.1)	9(12.9)		
**Marriage**				6.53	0.011
Married	785(88.6)	604(76.9)	181(23.1)		
Unmarried/Divorced /Widowed	101(11.4)	66(65.3)	35(34.7)		
**Average monthly income**					
<3000	727(82.1)	551(75.8)	176(24.2)	0.064	0.801
≥ 3000	159(17.9)	119(74.8)	40(25.2)		
**Attitude(classification by median)**				30.434	<0.001
High	617(69.6)	499(80.9)	118(19.1)		
Low	269(30.4)	171(63.6)	98(36.4)		
**Perceived behavioural control (classification by median)**				12.073	0.001
High	669(75.5)	525(78.5)	144(21.5)		
Low	217(24.5)	145(66.8)	72(33.2)		
**Subjective norm** **(classification by median)**				31.017	<0.001
High	650(73.4)	523(80.5)	127(19.5)		
Low	236(26.6)	147(62.3)	89(37.7)		

### Multi-factor regression analysis

Using a stepwise approach, we fitted an unconditional binary logistic model incorporating significant variables (*p* < 0.05) from univariate analysis. Factors influencing hypertensive patients’ willingness to vaccinate (*p* < 0.05), include: (1) Age: for the 45–49 age group (OR = 2.030, 95% CI: 1.306–3.157) and the 18–44 age group (OR = 5.007, 95% CI: 1.766–14.200) compared to 60+; (2) Attitude: high vs. low score (OR = 1.919, 95% CI: 1.352–2.724); (3) Behavioral Control: high vs. low score (OR = 1.604, 95% CI: 1.120–2.297); and (4) Subjective Norms: high vs. low score (OR = 1.782, 95% CI: 1.237–2.568).

### Reliability and validity test

Results indicated that the factor loadings for each latent variable in the model were robust. Specifically, Attitude had loadings of 0.833 and 0.860, Subjective Norm had loadings of 0.784, 0.929, 0.936, and 0.941, Perceptual Behavior Control showed loadings of 0.821, 0.838, and 0.910, Intention demonstrated loadings of 0.836 and 0.925, and Perception of Severity revealed loadings of 0.831 and 0.841. These strong loadings effectively captured the latent variables in the model.

The Cronbach α coefficients for the observation variables ranged between 0.812 and 0.943, consistently exceeding 0.8. Additionally, the Composite Reliability (CR) for each dimension fluctuated between 0.823 and 0.944, with all scores above 0.7. Furthermore, the Average Variance Extracted (AVE) spanned from 0.699 to 0.810, surpassing the 0.5 threshold across all dimensions. Please refer to Table 2 for detailed data. See Table 2.

In terms of discriminant validity, the square root of AVE for each dimension was greater than the correlation coefficient of other dimensions, indicating that the scale possesses good discriminant validity, see Table 3. Furthermore, all Heterotrait-Monotrait Ratio (HTMT) values were less than 0.85, further confirming the good discriminant validity of the scale [10]. See Table 4.

**Table 2:** Reliability and validity analysis of COVID-19 vaccination intention in TPB model

Dimension	Observation variable	Average value	Standard deviation	Factor loading	Cronbach α	AVE	CR
**Subjective norm**	Family	4.55	0.854	0.784	0.943	0.810	0.944
	People around	4.50	0.892	0.929			
	Medical staff	4.54	0.867	0.936			
	Nation	4.58	0.849	0.941			
**Attitude**	Protective effect	4.51	0.798	0.833	0.812	0.717	0.835
	Safety	4.49	0.847	0.86			
**Perceived behavioural control**	Convenient appointment	4.12	1.224	0.91	0.891	0.735	0.892
	Distance of location	3.96	1.343	0.838			
	Vaccination times	4.06	1.251	0.821			
**Intention**	Care Protection Rate	4.63	1.015	0.925	0.869	0.777	0.874
	Get vaccinated asap	4.40	1.142	0.836			
**Risk perception**	Burden	4.36	1.158	0.831	0.823	0.699	0.823
	Life	4.36	1.135	0.841			

**Table 3:** Correlation analysis of COVID-19 vaccination intention variables

Dimension	Attitude	Subjective norm	Risk perception	Intention	Perceived behavioural control
**Attitude**	0.851				
**Subjective norm**	0.362***	**0.9**			
**Risk perception**	0.508***	0.231***	**0.836**		
**Intention**	0.284***	0.379***	0.098*	**0.881**	
**Perceived behavioural control**	0.111**	0.357***	0.023	0.129***	**0.857**

*NoteP*<0.05 *, *P*<0.001 **, *P*<0.001 ***, the numbers in bold are the square root value of AVE in each dimension

**Table 4:** Heterotrait-Monotrait Ratio (HTMT) analysis

Dimension	Attitude	Subjective norm	Risk perception	Intention
**Subjective norm**	0.38			
**Risk perception**	0.509	0.246		
**Intention**	0.286	0.375	0.088	
**Perceived behavioural control**	0.108	0.352	0.022	0.139

### Structural equation model fitting

Based on the assumptions of H1-H7 above, we developed a structural equation model. The model fit statistics were χ2/df = 2.305, Comparative Fit Index (CFI) = 0.989, Tucker Lewis Index (TLI) = 0.985 and Root Mean Square Error of Approximation (RMSEA) = 0.038, which satisfied the requirements of “χ2/df < 3, CFI ≥ 0.90, TLI ≥ 0.90, Root Mean Square Error of Approximation (RMSEA) ≤ 0.08”, indicating that the established model fit appropriately [11].

### Structural equation modelling analysis

This model showed that there were three pathways that significantly influenced behavioral attitudes of people with hypertension as follows: (1) Knowledge (path coefficient = 0.127, CR = 6.887, *P* < 0.001); (2) Risk perception (path coefficient = 0.305, CR = 10.607, *P* < 0.001); (3) Subjective Norms (path coefficient = 0.175, CR = 5.752, *P* < 0.001), all three pathways had a positive effect relationship on Attitude, with risk perception having the highest positive effect; whereas Perceptual Behavioural Control (path coefficient = -0.011, CR = -0.505, *P* = 0.614) had a non-significant effect on attitude. Hypotheses H1, H2 and H3 were tested and the hypotheses were validated, while H4 did not pass the test.

The following two pathways had a significant effect on vaccination intention in people with hypertension: Subjective Norms (path coefficient = 0.361, CR = 8.049, *p* < 0.001) and Attitude (path coefficient = 0.253, CR = 4.447, *p* < 0.001), both of which had a positive relationship on vaccination intention, with Subjective Norms having a slightly higher positive effect than Attitudes; Perceptual Behavioural Control (path coefficient = -0.004, CR = -0.127, *P* = 0.899) had no significant effect on attitude. Therefore, hypotheses H5 and H6 hold and hypothesis H7 does not pass the model test. See Table 5.

The mediation analysis revealed the following three pathways in which attitude as a mediating factor significantly influenced the willingness to vaccinate in people with hypertension: (1) Knowledge → Attitude → Intention (indirect path coefficient = 0.032, LLCI = 0.014, ULCI = 0.058), (2) Risk perception → Attitude → Intention (indirect path coefficient = 0.077, LLCI = 0.038, ULCI = 0.124), (3) Subjective norms → Attitudes → Intention (indirect path coefficient = 0.044, LLCI = 0.019, ULCI = 0.087). And in both paths (1) and (2), Attitudes were used as complete mediators. See Table 6.

**Table 5:** Regression coefficient table of the model

Dimension	Estimate	S.E.	C.*R*.	*P*
Subjective norm → Attitude	0.172	0.03	5.752	***
Risk perception → Attitude	0.305	0.029	10.607	***
Knowledge score → Attitude	0.127	0.018	6.887	***
Perceived behavioural control → Attitude	-0.011	0.022	-0.505	0.614
Subjective norm → Intention	0.361	0.045	8.049	***
Perceived behavioural control → Intention	-0.004	0.032	-0.127	0.899
Attitude → Intention	0.253	0.057	4.447	***

Note: *P*<0.05 *, *P*<0.001 **, *P*<0.001 ***,

**Table 6:** Analysis of mediation effects

Indirect path	Coeff.	SE	ULCI	LLCI
Knowledge → Attitude → Intention	0.032	0.011	0.058	0.014
Subjective norm → Attitude → Intention	0.044	0.017	0.087	0.019
Risk perception → Attitude → Intention	0.077	0.022	0.124	0.038

## Discussion

Hypertension is a common chronic disease in China, people living with hypertension are more likely to develop severe complications after being infected with COVID-19. The present study found that 75.6% were willing to receive COVID-19 vaccination among people with hypertension. This result is lower than the findings of previous studies conducted in the general population in China, which reported a willingness rate of around 86.8% [12]. Some hypertensive patients are concerned about the adverse effects of vaccination on blood pressure, while others are unaware that hypertensive patients can also be vaccinated, resulting in a lower willingness to get vaccinated compared to the general population.

The TPB model we established tested hypotheses H1 to H7, revealing that Knowledge, Risk perception, and Subjective Norms all favorably influenced Attitude. Notably, these three components had a significant impact on the behavioral attitudes of hypertensive participants, with Risk perception demonstrating the most profound positive effect. Additionally, both Subjective Norms and Attitude played a crucial role in shaping vaccination intention among hypertensive individuals. These two factors were directly correlated with vaccination intention, where Subjective Norms demonstrated a marginally stronger positive influence compared to Attitudes. These findings align with prior research outcomes [13–16], indicating that targeted interventions designed to enhance vaccination attitudes and subjective norms in hypertensive patients can significantly boost their propensity to accept the COVID-19 vaccine [17].

The mediation analysis conducted in this study further discovered that among the factors influencing hypertensive patients’ willingness to get vaccinated, including vaccination knowledge, risk perception, and subjective norms, there are three paths that affect their willingness through the mediating factor of Attitude, as follows: (1) Knowledge → Attitude → Intention (2) Risk perception → Attitude → Intention (3) Subjective norms → Attitudes → Intention. Notably, in both paths (1) and (2), Attitudes served as complete mediators. These findings align with previous research outcomes [16, 18, 19]. Therefore, enhancing COVID-19 vaccination knowledge dissemination, risk perception, and strengthening the influence of their social networks to facilitate attitude change among hypertensive patients towards COVID-19 vaccination, is an effective way to increase their willingness to get vaccinated.

The limitation of this study stems from its cross-sectional design, thereby constraining the extrapolation of its findings. However, the results of Cronbach’s alpha coefficients, Composite Reliability, Discriminant Validity, and Factor Loadings of the research model are all satisfactory, indicating good performance both in internal stability and consistency.

In conclusion, the willingness of people with hypertension to receive COVID-19 vaccination is not high enough to achieve herd immunity. The TPB is a useful theoretical framework for understanding their vaccination behavior. To effectively promote a change in hypertensive patients’ willingness to get vaccinated, efforts should be concentrated on strengthening subjective norms, increasing vaccination knowledge, and, crucially, shaping positive behavioral attitudes. Thereby increasing vaccination rates and minimizing the impact of the COVID-19 epidemic on hypertensive individuals.

## Data Availability

The used or analyzed data during the current study is available from the corresponding author on reasonable request.

## Abbreviations

COVID: 19-Coronavirus disease 2019
TPB: Theory of Planned Behavior
CR: Composite Reliability
AVE: Average Variance Extracted
CFI: Comparative Fit Index
TLI: Tucker Lewis Index
RMSEA: Root Mean Square Error of Approximation
